# LB18. Healthcare Utilization for Acute Respiratory Illness by Race/Ethnicity across Ambulatory, Emergency, and Hospital Settings

**DOI:** 10.1093/ofid/ofab466.1654

**Published:** 2021-12-04

**Authors:** Alexandra M Mellis, Matthew Gilmer, Carrie Reed

**Affiliations:** 1 Centers for Disease Control and Prevention, Atlanta, Georgia; 2 GDIT, Atlanta, Georgia; 3 CDC, Atlanta, Georgia

## Abstract

**Background:**

Given the disproportionate impact of COVID-19 among racial/ethnic minority groups across the United States on emergency visits, hospitalizations, and deaths, we examined healthcare utilization more broadly for acute respiratory illness (ARI across healthcare settings by racial/ethnic group.

**Methods:**

Using data on 33,992,254 unique nonpharmacy healthcare encounters from the IBM Explorys Electronic Health Record database from January 1, 2020–May 1, 2021, across healthcare settings (ambulatory care or telehealth, emergency department, and hospitalizations) with nonmissing bridged racial/ethnic data. Encounters were classified as ARI based on ICD-10 and SNOMED codes and aggregated by month and US Census region. We estimated the population denominator as the total number of persons by bridged racial/ethnic group with encounters recorded during 2019. We both estimated the rate of ARI visits per 100,000 persons across healthcare settings and the rate ratio of ARI visits to non-ARI visits. We performed comparisons of these values by race/ethnicity, taking White persons as referent, using Poisson generalized estimating equations clustered within geographic regions.

**Results:**

A total of 244,137 (6.5% of 3,745,135) hospitalizations, 237,873 (18% of 1,305,474) emergency visits, and 1,636,383 (5.7% of 28,941,645) ambulatory visits were associated with ARIs. We observed similar rates of ARI visits across race/ethnicity groups in all settings combined and in ambulatory settings, but higher rates of ARI hospitalization among Hispanic persons (IRR [95% CI]: 2.5 [1.7–3.7]) and higher rates of ARI emergency department visits among Black persons (2.5 [1.9–3.2]) (Figure). We also observed differences in the relative proportion of care received for ARI vs. other visits types by setting, for example with Black persons utilizing higher rates of hospital visits for ARI vs non-ARI care (2.2 [1.7–2.7]) but lower rates of ambulatory care for ARI (0.9 [0.7–0.96]).

ARI Visits Per 100k Persons

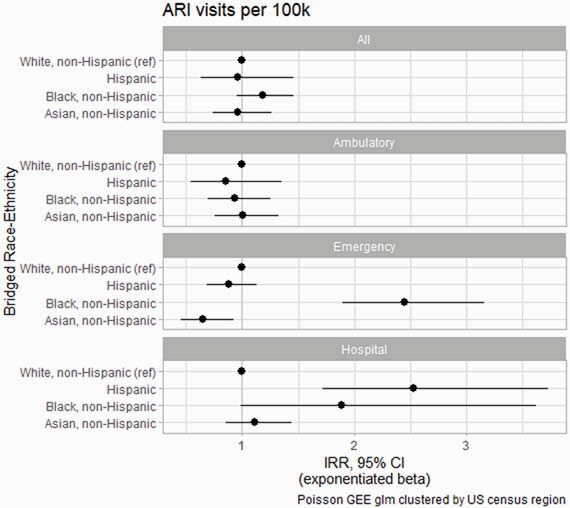

**Conclusion:**

Population rates of ARI visits and relative proportions of ARI vs. non ARI visits differed between racial/ethnic groups by setting. Understanding how utilization of care varies for ARI across settings can inform future monitoring efforts for health equity.

**Disclosures:**

**All Authors**: No reported disclosures

